# Prediction of Severity in Acute Pancreatitis

**DOI:** 10.1155/1991/78406

**Published:** 1991

**Authors:** Waldemar Uhl, Hans Günther Beger

**Affiliations:** Department of General Surgery University of Ulm Steinhövelstrasse 9 Ulm W-7900 Germany


*HPB Surgery*, 1991, Vol. 5, pp. 61–75
Reprints available directly from the publisher
Photocopying permitted by license only

© 1991 Harwood Academic Publishers GmbH
Printed in the United Kingdom

### HPB INTERNATIONAL

EDITORIAL & ABSTRACTING SERVICE JOHN TERBLANCHE, EDITOR
Department of Surgery,
Medical School · Observatory 7925
Cape Town · South Africa
Telephone: (021) 47-1250 Ext. 229
Telefax (021) 47-8955

### PREDICTION OF SEVERITY IN ACUTE PANCREATITIS

### ABSTRACT

*Fan, ST, Choi, TK, Lai, ECS and Wong, J. (1989) Prediction of severity of acute pancreatitis: an alternative approach. Gut;* **30**, 1591-1595

**Admission laboratory data of 203 patients suffering from acute pancreatitis were analysed to search for a simpler method of prediction of severity than the traditional multifactor prognostic scoring system. By discriminant analysis, admission serum urea and plasma glucose were identified to be factors with independent significance in predicting severity. If the presence of either factor higher than the cutoff point (urea >7.4 mmol/l, glucose >11.0 mmol/l) was considered as an indication of severe disease, then the sensitivity of this method was 75.0%, specificity 80.3% and the accuracy 79.3%. The predictive ability of this method was comparable with the Glasgow multifactor scoring system when the latter was also used to grade severity of our patients. It has the advantage, however, of simplicity and the ability of predicting severity at the time of admission.**

### PAPER DISCUSSION

KEY WORDS: Pancreatitis, acute

According to clinical and morphological criteria acute pancreatitis can be divided into two subgroups. On the one hand, it is a self-limiting disease, morphologically characterized by an interstitial edema within and outside the pancreatic gland, sometimes with additional peripancreatic fatty tissue necrosis and linked to a mortality rate of below 1%. On the other hand, there is the morphologically characterized hemorrhagic necrotizing form of acute pancreatitis with high morbidity and mortality rates, despite new therapeutic procedures introduced recently. In the literature a mortality rate of 10 to 15% for the clinically severe form of acute pancreatitis is given, mainly caused by the bacterial contamination of the necroses, followed by septic complications and multiple organ failure in the late phase of the disease[1,2,3].

A precise and early discrimination between these two courses of acute pancreatitis has been the aim of many clinical studies in the past[4,5]. Among the clinical/ laboratory staging systems the Ranson score, comprising 11 different factors, is most frequently used worldwide[6]. The study by Fan *et al.* also deals with the early prediction of the degree of severity of acute pancreatitis. The laboratory analysis of 17 parameters revealed serum urea and plasma glucose as best suitable for the prediction of the clinical severity of acute pancreatitis, with an accuracy rate of about 80%. Unfortunately the authors do not supply any morphological data, comparable to those of other studies today. In this respect contrast-enhanced computed tomography must be mentioned particularly, as this method has established itself worldwide as the "gold standard" in the staging of acute pancreatitis, with a high degree (85-90%) of sensitivity and specificity in the detection of necrotizing pancreatitis[7,8]. In the present paper an acute pancreatitis was grouped as "severe" if the outcome was lethal, if more than one local or systemic complication occurred, or if an emergency operation was necessary, due to "biliary infection" or a complication of acute pancreatitis. According to this definition the 203 included patients were subdivided into a group of 163 with a mild, and another group of 40 with a severe form of the disease. However, it is at least doubtful whether patients with an emergency operation or with cholangitis, an exploration of the biliary duct system or a perforated duodenal ulcer can be rightly included in the "severe" group. *Per se* these diseases have nothing to do with an acute pancreatitis or with a complication directly linked to it.

As early as 1974 Ranson and his co-workers[6] inaugurated a staging system for acute pancreatitis on the basis of objective biochemical parameters and the patients' age. From a computerized analysis, consisting of 43 parameters, they singled out 11, the amount of prognostic indices correlating directly with morbidity and mortality. Unfortunately the authors do not give the so-called Ranson-points for the two groups of acute pancreatitis they deal with, inspite of the fact that this scoring system has gained general acceptance in the literature with regard to stratification and comparability. They must be reproached with imitating Ranson's work, on the basis of 17 parameters only. As a reason for their prospective analysis the authors cite three disadvantages of Ranson's system: firstly, it comprises too many factors; secondly, it necessitates an observation period of 48 hours for an assessment; and thirdly, some parameters are considerably influenced by the applied therapy. However, this last argument is particularly true for the serum plasma glucose and serum urea levels. The authors examined the same 17 parameters 48 hours later, but do not give the results; it must be supposed therefore, that still more laboratory parameters play a role in the discrimination between mild and severe acute pancreatitis.

Apart from the development of new scoring systems, as for example the Glasgow system[10], several single biochemical parameters have been examined recently[5,11-17], in order to achieve an early prognosis of acute pancreatitis. In prospective clinical trials in which patients with acute pancreatitis were strictly subdivided according to morphological criteria, high accuracy rates in the detection of pancreatic necroses were achieved by an evaluation of C-reactive protein, PMN-elastase, lactate dehydrogenase (LDH), catalytic phospholipase A2-activity (PLA2) and $\alpha$ 2-macroglobulin[5,12,15,17].

In an analysis of the Department of General Surgery in Ulm, the calculated accuracy rates for the correct prognosis of the degree of severity (morphology) of acute pancreatitis within the first 5 pre-operative days after onset of the disease were 86% for CRP (cut-off-level >120 mg/l), 84% for PMN-elastase (>120 $\mu$g/l), 82% for LDH (>270 U/l), 77% for the phospholipase A2-activity (>3.5U/l) and 72% for $\alpha$2-macroglobulin (<1.5 g/l; 12, 15, 17). Thus, the first three parameters exceed by far the accuracy rate of the combined serum urea and plasma glucose levels, given by the authors, for the discrimination between mild and severe acute pancreatitis.

In summary it must be admitted, that the present paper, based on a Chinese patient collective and worked out according to clinical criteria, is an interesting prospective analysis concerning the discrimination between mild and severe forms of acute pancreatitis. However, it does not supply new insights to the clinically active colleagues.

### REFERENCES

1. Warshaw, A.L. (1980) A guide to pancreatitis. *Compr.Ther.*, **6**, 49–55
2. Beger, H.G., Krautzberger, W., Bittner, R., Block, S. and Büchler, M. (1985) Results of surgical treatment of necrotizing pancreatitis. *World J.Surg.*, **90**, 972–979
3. Beger, H.G., Krautzberger, W., Bittner, R., Büchler, M. and Block, S. (1986) Bacterial contamination of pancreatic necrosis. A prospective clinical study. *Gastroenterology*, **91**, 433–438
4. Beger, H.G. and Büchler, M. (1986) Decision-making in surgical treatment of acute pancreatitis: operative or conservative management of necrotizing pancreatitis? *Theor.Surg.*, **1**, 61–68
5. Büchler, M. (1991) Objectification of the severity of acute pancreatitis. *Hepatogastroenterol*, **38**, 101–108
6. Warshaw, A.L. and Jin, G. (1985) Improved survival in 45 patients with pancreatic abscess. *Ann.Surg.*, **202**, 408–417
7. Ranson, J.H.C., Rifkind, K.M., Roses, D.F., Fink, S.D., Eng, K. and Spencer, F.C. (1974) Prognostic signs and the role of operative management in acute pancreatitis. *Surg.Gynecol.Obstet.*, **139**, 69–81
8. Kivisaari, L., Somer, K., Standertskjöld-Nordenstam, C.G., Schröder, T., Kivilaakso, E. and Lempinen, M. (1984) A new method for the diagnosis of acute hemorrhagic necrotising pancreatitis using contrast-enhanced CT. *Gastrointest.Radiol.*, **9**, 27–30
9. Block, S., Maier, W., Clausen, C., Bittner, R., Büchler, M. and Beger, H.G. (1986) Identification of pancreas necrosis in severe acute pancreatitis. *Gut*, **27**, 1035–1042
10. Balhazar, E.J. (1989) CT diagnosis and staging of acute pancreatitis. *Rad.Clin.North Am.*, **27**, 19–37
11. Osborne, D.H., Imrie, C.W. and Carter, D.C. (1981) Biliary surgery in the same admission for gallstone-associated pancreatitis. *Br.J.Surg.*, **68**, 758–761
12. Mayer, A.D., McMahon, M.J., Bowen, M. and Cooper, E.H. (1984) C-reactive protein: an aid to assessment and monitoring of acute pancreatitis. *J.Clin.Pathol.*, **37**, 207–211
13. Büchler, M., Malfertheiner, P., Schoetensack, C., Uhl, W. and Beger, H.G. (1986) Sensitivity of antiproteases, complement factors and C-reactive protein in detecting pancreatic necrosis. Results of a prospective clinical study. *Int.J.Pancreatol*, **1**, 227–235
14. Puolakkainen, P., Valtanen, V., Paananen, A. and Schröder, T. (1987) C-reactive protein and serum Phospholipase A2 in the assessment of severity of acute pancreatitis. *Gut*, **28**, 764–771
15. Wilson, C., Heads, A., Shenkin, A. and Imrie, C.W. (1989) C-reactive protein, antiproteases and complement factors as objective markers of severity in acute pancreatitis. *Br.J.Surg.*, **76**, 177–181

16. Büchler, M., Malfertheiner, P., Schädlich, H., Nevalainen, T.J., Friess, H. and Beger, H.G. (1989) Role of Phospholipase A2 in human acute pancreatitis. *Gastroenterology*, **97**, 1521–1526
17. Gudgeon, A.M., Heath, D., Hurley, P., Shenkin, A., Jehanli, A., Patel, G., Wilson, C., Austen, B.M. and Imrie, C.W. (1988) Trypsinogen activation peptide (TAP) assay in severity assessment of acute pancreatitis. *Pancreas*, **3**, 598 (abstract)
18. Uhl, W., Büchler, M., Malfertheiner, P., Martini, M. and Beger, H.G. (1991) PMN-Elastase in comparison with CRP, antiproteas and LDH as indicators of necrosis in human acute pancreatitis. *Pancreas*, **6**, 253–259

Waldemar Uhl
Hans Günther Beger
Department of General Surgery
University of Ulm
Steinhövelstrasse 9
W-7900 Ulm
Germany

### PANCREATIC DRAINAGE INTO THE STOMACH AFTER PANCREATIC RESECTION

### ABSTRACT

*Delcore, R., Thomas, J.H., Pierce, G.E. and Hermreck, A.S. (1990) Pancreatogastrostomy: A safe drainage procedure after pancreatoduodenectomy. Surgery;* **108**: 641-647

**The purpose of this study was to evaluate the role of pancreaticogastrostomy as an alternative method of restoring pancreaticointestinal continuity after pancreaticoduodenectomy. Since 1975, 45 patients have undergone pancreaticogastrostomy after pancreaticoduodenectomy at our institution. Pancreaticoduodenectomy was performed for pancreatic carcinoma (24 patients), ampullary carcinoma (8 patients), duodenal carcinoma (4 patients), common bile duct carcinoma (4 patients), pancreatic islet cell carcinoma (1 patient), trauma (1 patient), extensive colon carcinoma (1 patient), chronic pancreatitis (1 patient), and gastroduodenal artery aneurysm (1 patient). There was one operative death, for an overall operative mortality rate of 2%, and seven patients had major postoperative complications, for an overall morbidity rate of 15%. No pancreatic anastomotic leaks or other complications related to the pancreaticogastrostomy occurred. Twenty-four patients have died of recurrent carcinoma, with a mean survival of 25 months (range, 5 to 66 months), and 20 patients are alive and well, with a mean follow-up of 27 months (range, 2 to 106 months). Eight of these patients are alive 2 or more years after operation and four do not have exocrine pancreatic insufficiency. This experience confirms that pancreaticogastrostomy is a safe method of pancreatic drainage after pancreaticoduodenectomy and suggests that it may have technical advantages and therefore merits more widespread application. (Surgery 1990; 108:641-7.)**

